# Long term predictive values of cytology and human papillomavirus testing in cervical cancer screening: joint European cohort study

**DOI:** 10.1136/bmj.a1754

**Published:** 2008-10-13

**Authors:** Joakim Dillner, Matejka Rebolj, Philippe Birembaut, Karl-Ulrich Petry, Anne Szarewski, Christian Munk, Silvia de Sanjose, Pontus Naucler, Belen Lloveras, Susanne Kjaer, Jack Cuzick, Marjolein van Ballegooijen, Christine Clavel, Thomas Iftner

**Affiliations:** 1 Lund University, Medical Microbiology, University Hospital MAS, 205 02 Malmö, Sweden; 2 Department of Public Health, Erasmus MC, PO Box 2040, 3000 CA Rotterdam, Netherlands; 3 CHU Reims, Service d’Anatomie Pathologique, INSERM UMR-S 903, Laboratoire Pol Bouin, Reims, France; 4 Department of Obstetrics and Gynaecology, Teaching Hospital Wolfsburg, Germany; 5 Cancer Research UK, Wolfson Institute of Preventive Medicine, London; 6 Institute of Cancer Epidemiology, Danish Cancer Society, DK-2100, Copenhagen, Denmark; 7 Catalan Institute of Oncology, Hospital Duran i Reynals, Barcelona, Spain; 8 Gynecologic Clinic, Rigshospitalet, Copenhagen University Hospital, Denmark; 9 CIBER Epidemiologia y Salud Pública (CIBERESP), Spain; 10 Section of Experimental Virology, Institute of Medical Virology, University Hospital of Tübingen, Germany

## Abstract

**Objective** To obtain large scale and generalisable data on the long term predictive value of cytology and human papillomavirus (HPV) testing for development of cervical intraepithelial neoplasia grade 3 or cancer (CIN3+).

**Design** Multinational cohort study with joint database analysis.

**Setting** Seven primary HPV screening studies in six European countries.

**Participants** 24 295 women attending cervical screening enrolled into HPV screening trials who had at least one cervical cytology or histopathology examination during follow-up.

**Main outcome measure** Long term cumulative incidence of CIN3+.

**Results** The cumulative incidence rate of CIN3+ after six years was considerably lower among women negative for HPV at baseline (0.27%, 95% confidence interval 0.12% to 0.45%) than among women with negative results on cytology (0.97%, 0.53% to 1.34%)). By comparison, the cumulative incidence rate for women with negative cytology results at the most commonly recommended screening interval in Europe (three years) was 0.51% (0.23% to 0.77%). The cumulative incidence rate among women with negative cytology results who were positive for HPV increased continuously over time, reaching 10% at six years, whereas the rate among women with positive cytology results who were negative for HPV remained below 3%.

**Conclusions** A consistently low six year cumulative incidence rate of CIN3+ among women negative for HPV suggests that cervical screening strategies in which women are screened for HPV every six years are safe and effective.

## Introduction

Cytological screening has reduced the incidence of cervical cancer in countries with organised screening,^1^ but in Europe in 1995 there were still an estimated 68 000 incident cases.^2^ Cytology has limited reproducibility,^3^ and both meta-analyses and pooled analyses of cross sectional studies have established that tests for human papillomavirus (HPV) have higher sensitivity than cytology in detecting high grade cervical intraepithelial lesions (CIN)^4^ ^5^ and that combined HPV and cytology testing has high negative predictive values for CIN.^6^ ^7^ ^8^ Cost effectiveness modelling of screening strategies, however, depends greatly on reliable and generalisable estimates of the longitudinal, long term predictive values of testing. The long term negative predictive value is the main determinant of the safe screening interval to use, a key factor for the cost efficiency of a screening programme. The long term positive predictive value is an important measure of the extent of unnecessary procedures induced by screening, another major factor in evaluations of cost efficiency. As low and moderate grades of CIN often regress, predictive values to be used for modelling should ideally use CIN grade 3 or cancer (CIN3+) as the outcome.^9^

Several randomised controlled trials are currently being conducted to compare primary screening based on HPV detection with conventional cytology screening.^10^ ^11^ ^12^ ^13^ ^14^ ^15^ ^16^ Data from these trials indicate that HPV based screening results in detection of more high grade CIN lesions (a higher sensitivity) but a reduced specificity compared with cytology based screening. The randomised trials found that the increased sensitivity for CIN3+ is not merely overdiagnosis as there is a correspondingly lower incidence of CIN3+ in the future,^11^ ^12^ ^13^ ^14^ further establishing the validity of using CIN3+ as end point in studies of HPV based cervical screening.

Most of the cohort studies and the randomised trials, however, have observed only limited numbers of cases of CIN3+ on longer term follow-up, resulting in reduced statistical power for estimating the critical factor for deciding the appropriate screening interval: the rate of CIN3+ among women with negative results at screening. Furthermore, clinical and diagnostic practices vary between European countries, and different studies have often used different methods for evaluation, making meta-analyses difficult.

To obtain large scale and generalisable data on long term predictive values for CIN3+, we obtained primary data from seven HPV screening studies in six EU countries, each investigating the predictive value of primary HPV screening for future CIN3+; assessed variability between studies; and estimated the overall long term predictive values for CIN3+.

## Methods

The seven prospective HPV studies supplied data to a common database for joint statistical analysis. Table 1 provides details of the design of the seven studies, inclusion and exclusion criteria, setting, and location[Table tbl1]. All studies used routine cytology as currently practised in their country. Table 1 also describes the different HPV tests used for each country. For all studies the people executing either test were unaware of the results of the other test. Comparability and reproducibility of the two main HPV tests used (hybrid capture II and GP5+/6+ polymerase chain reaction) was evaluated with κ statistics.^17^ Recruitment was consecutive and data collection prospectively planned.

**Table 1 tbl1:** Study characteristics of seven European human papillomavirus (HPV) screening studies

Study	No initially screened	No analysed*	Age	Entry criteria	HPV test	Follow-up†	Histology
Germany- Hannover	4699	4107	≥30	No history of abnormal smear result, CIN, or treatment for cervical disease in past year and not pregnant	HCII	If cytology+ or HPV+ immediate and annual colposcopy‡ for 5 years. 5% of cytology−/HPV− to colposcopy after 5 years	Blinded central review
Germany-Tubingen	672	670	≥30	No history of abnormal smear result, CIN, or treatment for cervical disease in past year and not pregnant	HCII	Cytology−/HPV− to new tests after 5 years and if either positive referred to colposcopy	Blinded central review
Sweden	6448	5671	32-38	Participating in organised screening	GP5+/6+ PCR	Cytology−/HPV+ invited for new test >1 year later, if persistent HPV+ referred to colposcopy. Similar number of women randomly referred to colposcopy. Database linked with regional pathology registries	Regional pathology labs§
Denmark	2287	2274	20-29	No current evidence of cervical neoplasia	HCII	Study data base linked with the National Pathology Registry	Regional pathology lab§
UK	2720	2322	≥35	No previous cervical treatment or abnormal smear result within past 3 years	SHARP-PCR, HCI, HCII	SHARP-PCR+ or cytology+ referred to colposcopy	Blinded central review
France	17 247	7935	No age limits	No abnormal smear result or untreated cervical lesion in past 2 years. Not HIV positive	HCII	If cytology−/HPV+ new tests after 6-12 months and if persistent HPV+ referred to colposcopy. 15% cytology−/HPV− referred to colposcopy	Blinded central review
Spain	2012	1316	Matched to general population	Population registry or attending screening	HCII	Follow-up tests 1 and 5 years later. Persistently HPV+ referred to colposcopy	Regional pathology lab§

HCII=hybrid capture II; CIN=cervical intraepithelial neoplasia; PCR=polymerase chain reaction.

*Women with at least one follow-up cytology or histology.

†Follow-up procedures performed in addition to routine clinical practice.

‡Assessment by colposcopy.

§Blinded to HPV status.

### Denmark

In 1993-5 women in the general population were enrolled in a prospective cohort study of the clinical course of HPV and cervical neoplasia. They were interviewed and underwent a cervical smear tests for cytology and cervical swabs for detection of HPV DNA with hybrid capture II.^18^ Each woman’s unique 10 digit personal identification number was linked to the national pathology data bank (a nationwide computerised pathology register containing all cytological and histological diagnoses) to allow follow-up. Data from women with negative results on both tests were supplied to the joint database.

### Germany: Hannover and Tübingen studies

In 1999-2000 women aged ≥30 were invited to the medical universities in Hannover or Tübingen for a prospective cohort study (HAT trial) on HPV screening.^6^ In Hannover women were followed with colposcopy every 6-12 months if they had a positive result on a hybrid capture 2 test or positive cytology at baseline. Women with negative results on both tests underwent annual cervical smear tests and 5% were referred for colposcopy five years later. In Tübingen, women with negative results on both tests at baseline were followed up with cytology and hybrid capture II test after five years; and if either test result was positive then they were referred for colposcopy.

### United Kingdom

The UK study included women attending routine screening in west London to evaluate HPV based screening in women aged ≥35 during 1994-7.^19^ DNA analysis was initially performed with the polymerase chain reaction/SHARP system to detect HPV types 16, 18, 31, 33, 54, 51, 52, 56, and 58. Women with positive results on HPV tests or abnormal cytology were referred for colposcopy. The samples were retrospectively analysed with hybrid capture I for the first half of the study and by hybrid capture II for the second half of the study. HPV results reported in our analyses are based on hybrid capture results. All women were followed up with the National Health Service computerised call/recall system, which records all smear tests and their results. In addition, all women were invited to a follow-up visit with HPV test and colposcopy, and 507 out of 2982 attended.

### France

Women who participated in biennial or triennial routine screening in 1997-2002 in Reims, France, were invited to participate in a study to evaluate HPV testing with hybrid capture II in cervical cancer screening.^20^ All women with abnormal results on cytology were referred for colposcopy. Women with a positive HPV test but normal cytology results were recalled after 6-12 months for a repeat cytological smear and HPV test. If a cytological abnormality was found or if the woman had a persistent HPV infection she was referred for colposcopy. Women with normal cytology results and a negative HPV test at baseline were followed with standard biennial or triennial cervical screening. A random 15% of women with negative results on both tests at baseline were also referred for colposcopy.

### Sweden

In 1997-2000 women aged 32-38 who took part in organised cervical screening in five regions of Sweden (Gothenburg, Malmö, Stockholm, Umeå, and Uppsala) were invited to participate in a randomised population based trial of primary HPV screening with general primer GP5+/6+ polymerase chain reaction.^21^ In the intervention arm, women with a positive HPV test were invited for a second cytology and HPV test at least a year later, together with a similar number of women randomly selected from the control arm. Women with persistent HPV infection, as well as a similar number of women from the control arm, were invited for colposcopy.^21^ Women with abnormal cytology were referred for colposcopy in accordance with established clinical algorithms. We included all women in the intervention arm and the randomly selected women from the control arm. All study participants were followed by registry linkages with comprehensive regional cytology and pathology registries by using unique personal identification numbers.

### Spain

In 1997-2001 women randomly selected from the general population of the Barcelona metropolitan area or who were attending one of nine family planning clinics for routine screening were enrolled.^22^ Both enrolment strategies performed frequency matching to the underlying general population. The study estimated the incidence and prevalence of genital HPV infection and evaluated the predictive value of cytology and HPV testing for future CIN at one and five years’ follow-up. HPV testing was done with hybrid capture II. Women from the general population were referred for colposcopy if they had abnormal cervical cytology or were persistently positive for HPV at the end of follow-up. Women from the family planning clinics were referred for colposcopy if they had abnormal cervical cytology or two consecutively positive HPV test results.

### Statistical analysis

From the joint cohort, we included only women with adequate cytology and HPV tests at baseline and with at least one follow-up cytological or histological test. We regarded abnormal cytology as the equivalent of atypical squamous cells of uncertain significance (ASCUS) or worse for all the participating studies. Women were followed up from the date of the baseline test. Incidence depends on the number of person years of follow-up, and, for a disease detectable by screening, follow-up requires the person to have attended screening. Therefore, we censored follow-up at the date of diagnosis of the CIN3+ lesion (CIN3 or invasive cancer, including squamous and adenocarcinoma) or at the last registered testing date.

Firstly, we estimated the specific cumulative incidence rate of CIN3+ by original baseline group (cytology−/HPV−, cytology−/HPV+, cytology+/HPV−, and cytology+/HPV+) for each country, with 95% confidence intervals, using the non-parametric Kaplan-Meier product limit estimator for log(hazard).^23^ Secondly, to determine whether lack of homogeneity between the different studies in the joint cohort influenced results we used comparative analysis of systematically drawn subsamples of the joint cohort, so called bootstrap analysis.^24^ The bootstrap stratified random subsample was constructed by drawing, with replacement, firstly from studies and then from individuals within studies. We constructed and analysed 1000 bootstrap replications in the same manner as the original country specific analysis and used the mean of these 1000 replicas as the pooled estimate of the cumulative incidence rate corrected for heterogeneity, with 2.5 and 97.5 centiles as estimates for the 95% confidence interval. As a measure for heterogeneity, we compared the original cohort specific 95% confidence intervals with those obtained from the multilevel bootstrap. This can be transformed into an estimate of the overdispersion parameter (or “scale” parameter), where 1 points to no heterogeneity among the studies and >1 points to increasing levels of heterogeneity.^25^ ^26^ Thirdly, we calculated the test performance indices for cytology alone, HPV test alone, and cytology and HPV test combined (at least one of the two positive). Because we did not have complete data for all four original baseline groups, we excluded studies from Denmark and Tübingen from these analyses. These indices were calculated using 2×2 tables based on the cumulative incidence rate at 72 months for the different baseline test combinations, weighted by the proportion (adjusted for heterogeneity) of each of these subgroups at baseline.^27^ The 95% confidence intervals around the indices were obtained by bootstrapping.^25^ All analyses used S-PLUS 6.0 Professional Release 1.

## Results

Out of 24 295 women included in the pooled analyses, 381 developed histologically confirmed CIN3+ during six years’ of follow-up (table 2). The positive predictive value for future CIN3+ was highest among women with abnormal cytology and positive HPV test at baseline (cytology+/HPV+) (cumulative incidence rate 34%, 95% confidence interval 26.8% to 45.4%) (fig 1). Women with normal cytology but positive HPV test (cytology−/HPV+) had a continuously increasing cumulative incidence rate of CIN3+, eventually reaching 10% (6.2% to 15.1%) after six years. Women with abnormal cytology and negative HPV test (cytology+/HPV−) had a cumulative incidence rate for CIN3+ of 2.7% (0.6% to 6.0%). Women with both normal cytology and negative HPV test (cytology−/HPV−) had a low risk of future CIN3+ (0.28%, 0.10% to 0.47%). We compared the cumulative incidence rate of CIN3+ after being cytology−/HPV− with that of CIN3+ for normal cytology alone and negative HPV test alone (fig 2). At six years of follow-up, the rate of CIN3+ was significantly lower among women negative for HPV (0.27%, 0.12% to 0.45%) than among women with negative cytology results (0.97%, 0.53% to 1.34%). By comparison, the rate of CIN3+ at the most commonly recommended screening interval in Europe (three years) was 0.51% (0.23% to 0.77%) for women with negative cytology results and 0.12% (0.03% to 0.24%) for women negative for HPV. At five and four years of follow-up, the rates were 0.25% (0.12% to 0.41%) and 0.19% (0.08% to 0.32%) for women negative for HPV compared with 0.83% (0.50% to 1.13%) and 0.69% (0.39% to 0.98%) for women with negative cytology results. There was little difference in rates for CIN3+ between women with negative results on both tests and women negative for HPV (fig 2). The rate for CIN3+ among women positive for HPV was lower than for women with abnormal results on cytology but increased continuously and gradually approached the rate of women with positive results on cytology (fig 3).

**Figure fig1:**
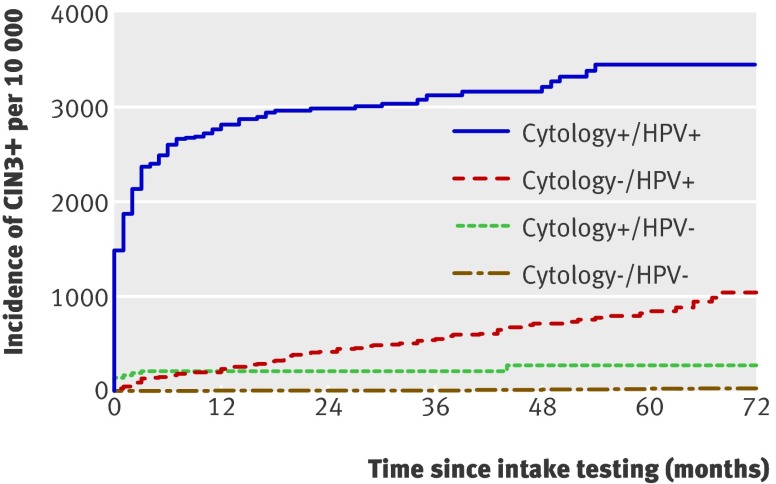
**Fig 1** Kaplan-Meier plots of cumulative incidence rate for CIN3+ for women according to baseline test results in the first 72 months of follow-up in all seven countries

**Figure fig2:**
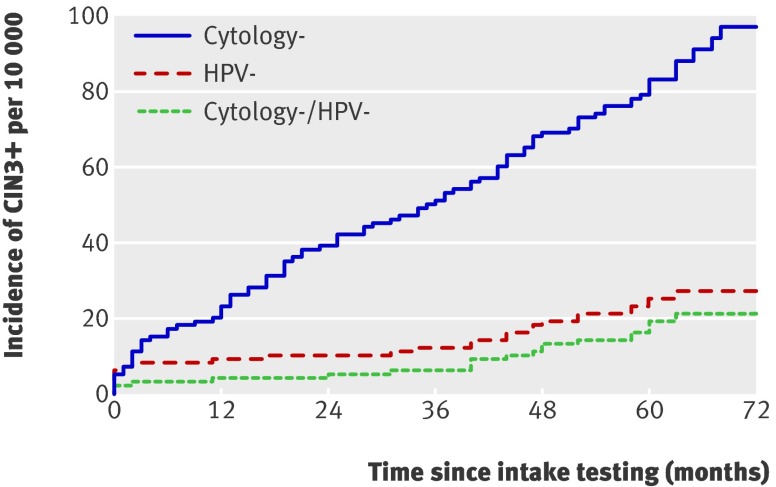
**Fig 2** Kaplan-Meier plots of cumulative incidence rate for CIN3+ for women according to baseline test results in first 72 months of follow-up, excluding Denmark and Tübingen

**Figure fig3:**
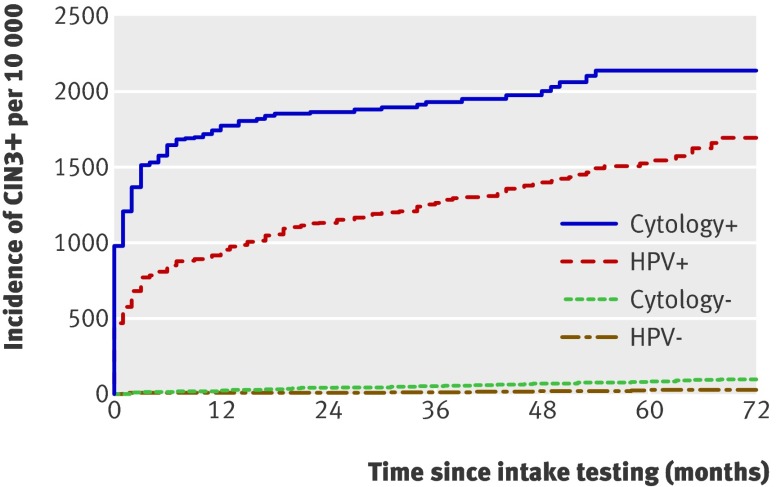
**Fig 3** Kaplan-Meier plots of cumulative incidence rate for CIN3+ for women according to baseline test reults in first 72 months of follow-up, excluding Denmark and Tübingen

**Table 2 tbl2:** Number of women in analysis, and number with cervical intraepithelial neoplasia grade 3 or cancer (CIN3+) diagnosed within 6 years of baseline

Baseline group	No at baseline*	No still in follow-up		No with CIN3+
		After 60 months	After 72 months	
Cytology−/HPV−	21 060	7019	4571	32
Cytology−/HPV+	1962	268	111	107
Cytology+/HPV−	436	89	46	10
Cytology+/HPV+	837	67	40	232
Total	24 295	7443	4778	381

*With at least one cytology or histology follow-up.

Analysis with an alternative outcome definition that included all high grade lesions (CIN grade 2 or worse; CIN2+) showed essentially similar results but was based on a higher number of cases (n=585). For example, at six years of follow-up, the cumulative incidence rate of CIN2+ was 0.67% (0.39% to 1.11%) among women negative for HPV and 1.76% (1.00% to 2.47%) among women with negative cytology results. The rates at three years of follow-up were 0.19% (0.07% to 0.38%) and 0.79% (0.43% to 1.16%), respectively.

As the prevalence of HPV infection is highly age dependent and as cytological performance also varies with age, we analysed positive and negative predictive values, sensitivity, and specificity of the screening tests stratified by age group (tables 3, 4, and 5) [Table tbl4] [Table tbl5]. The sensitivity and negative predictive value of cytology improved with age (table 3). Both cytology (table 3) and the HPV test (table 4) had higher specificity for women above 35 years but did not improve any further among women above 49.

**Table 3 tbl3:** Test characteristics for cytology (95% CI) at 72 months, by age (years), excluding data from Denmark and Tübingen

	Overall	>49	35-49	<35	P*	30-34†	<30†	P‡
Negative at baseline	20 078	3380	10 308	6390		982	2080	
CIN3+	127	9	48	70		14	21	
Positive at baseline	1273	161	557	555		111	330	
CIN3+	242	33	112	97		25	48	
Sensitivity	0.599 (0.348 to 0.683)	0.760 (0.504 to 0.903)	0.641 (0.506 to 0.747)	0.431 (0.137 to 0.615)	0.034	0.642 (0.511 to 0.773)	0.524 (0.389 to 0.669)	0.268
Specificity	0.954 (0.930 to 0.977)	0.964 (0.960 to 0.970)	0.959 (0.940 to 0.979)	0.937 (0.891 to 0.974)	0.546	0.924 (0.915 to 0.934)	0.875 (0.864 to 0.884)	<0.001
NPV	0.990 (0.987 to 0.995)	0.997 (0.995 to 0.999)	0.993 (0.990 to 0.997)	0.979 (0.965 to 0.990)	0.008	0.981 (0.971 to 0.990)	0.975 (0.960 to 0.987)	0.479
PPV	0.211 (0.102 to 0.291)	0.198 (0.048 to 0.915)	0.227 (0.113 to 0.342)	0.200 (0.087 to 0.269)	0.924	0.296 (0.191 to 0.403)	0.160 (0.123 to 0.197)	0.021

CIN3+=cervical intraepithelial neoplasia grade 3 or cancer; NPV=negative predictive value; PPV=positive predictive value.

*P value for trend.

†As only studies in France and Spain contained women <30 with complete test data, data for women aged 30-34 and <30 as well as P value for trend are restricted to these cohorts only.

‡P value for trend.

**Table 4 tbl4:** Test characteristics for human papillomavirus (95% CI) at 72 months, by age (years), excluding data from Denmark and Tübingen

	Overall	>49	35-49	<35	P*	30-34†	<30†	P‡
Negative at baseline	18 552	3169	9740	5643		876	1683	
CIN3+	30	4	16	10		2	4	
Positive at baseline	2799	372	1125	1.302		217	727	
CIN3+	339	38	144	157		37	65	
Sensitivity	0.896 (0.796 to 0.949)	0.850 (0.452 to 0.986)	0.895 (0.749 to 0.971)	0.886 (0.778 to 0.966)	0.896	0.900 (0.785 to 0.965)	0.641 (0.433 to 0.873)	0.044
Specificity	0.893 (0.929 to 0.942)	0.912 (0.872 to 0.957)	0.914 (0.861 to 0.944)	0.847 (0.747 to 0.926)	0.405	0.921 (0.914 to 0.929)	0.873 (0.861 to 0.882)	<0.001
NPV	0.997 (0.996 to 0.999)	0.999 (0.995 to 1.000)	0.998 (0.996 to 1.000)	0.994 (0.988 to 0.999)	0.180	0.997 (0.993 to 0.999)	0.987 (0.974 to 0.997)	0.064
PPV	0.171 (0.127 to 0.214)	0.106 (0.040 to 0.149)	0.166 (0.100 to 0.219)	0.196 (0.141 to 0.271)	0.106	0.254 (0.179 to 0.333)	0.129 (0.097 to 0.168)	0.008

CIN3+=cervical intraepithelial neoplasia grade 3 or cancer; NPV=negative predictive value; PPV=positive predictive value.

*P value for trend.

†As only studies in France and Spain contained women <30 with complete test data, data for women aged 30-34 and <30 as well as P value for trend are restricted to these cohorts only.

‡P value for trend.

**Table 5 tbl5:** Test characteristics for cytology and human papillomavirus (at least 1 positive), (95% CI) at 72 months, by age (years), excluding data from Denmark and Tübingen

	Overall	>49	35-49	<35	P*	30-34†	<30†	P‡
Both negative at baseline	18 116	3084	9502	5530		850	1640	
CIN3+	20	3	8	9		2	4	
Positive at baseline	3235	457	1363	1415		243	770	
CIN3+	349	39	152	158		37	65	
Sensitivity	0.921 (0.841 to 0.964)	0.897 (0.621 to 0.986)	0.935 (0.826 to 0.991)	0.993 (0.792 to 0.968)	0.695	0.954 (0.893 to 0.988)	0.845 (0.694 to 0.962)	0.113
Specificity	0.872 (0.810 to 0.925)	0.887 (0.850 to 0.934)	0.892 (0.837 to 0.931)	0.829 (0.729 to 0.914)	0.452	0.813 (0.800 to 0.830)	0.694 (0.666 to 0.714)	<0.001
NPV	0.998 (0.996 to 0.999)	0.999 (0.997 to 1.000)	0.999 (0.997 to 1.000)	0.994 (0.987 to 0.999)	0.126	0.997 (0.993 to 0.999)	0.988 (0.975 to 0.997)	0.097
PPV	0.147 (0.099 to 0.190)	0.089 (0.038 to 0.127)	0.144 (0.081 to 0.204)	0.178 (0.125 to 0.248)	0.071	0.224 (0.151 to 0.294)	0.122 (0.092 to 0.157)	0.016

CIN3+=cervical intraepithelial neoplasia grade 3 or cancer; NPV=negative predictive value; PPV=positive predictive value.

*P value for trend.

†As only studies in France and Spain contained women <30 with complete test data, data for women aged 30-34 and <30 as well as P value for trend are restricted to these cohorts only.

‡P value for trend.

The seven studies included in the pooled analyses had estimates of cumulative incidence rate for CIN3+ that were not significantly different among cytology−/HPV−, cytology−/HPV+, or cytology+/HPV− women (scale parameters: 2.48, 1.80, 2.23; P values 0.14, 0.36, 0.1). The cumulative incidence rate of CIN3+ among women with positive cytology and HPV test, however, was clearly different between studies (scale parameter 4.77; P=0.01) (fig 4).

**Figure fig4:**
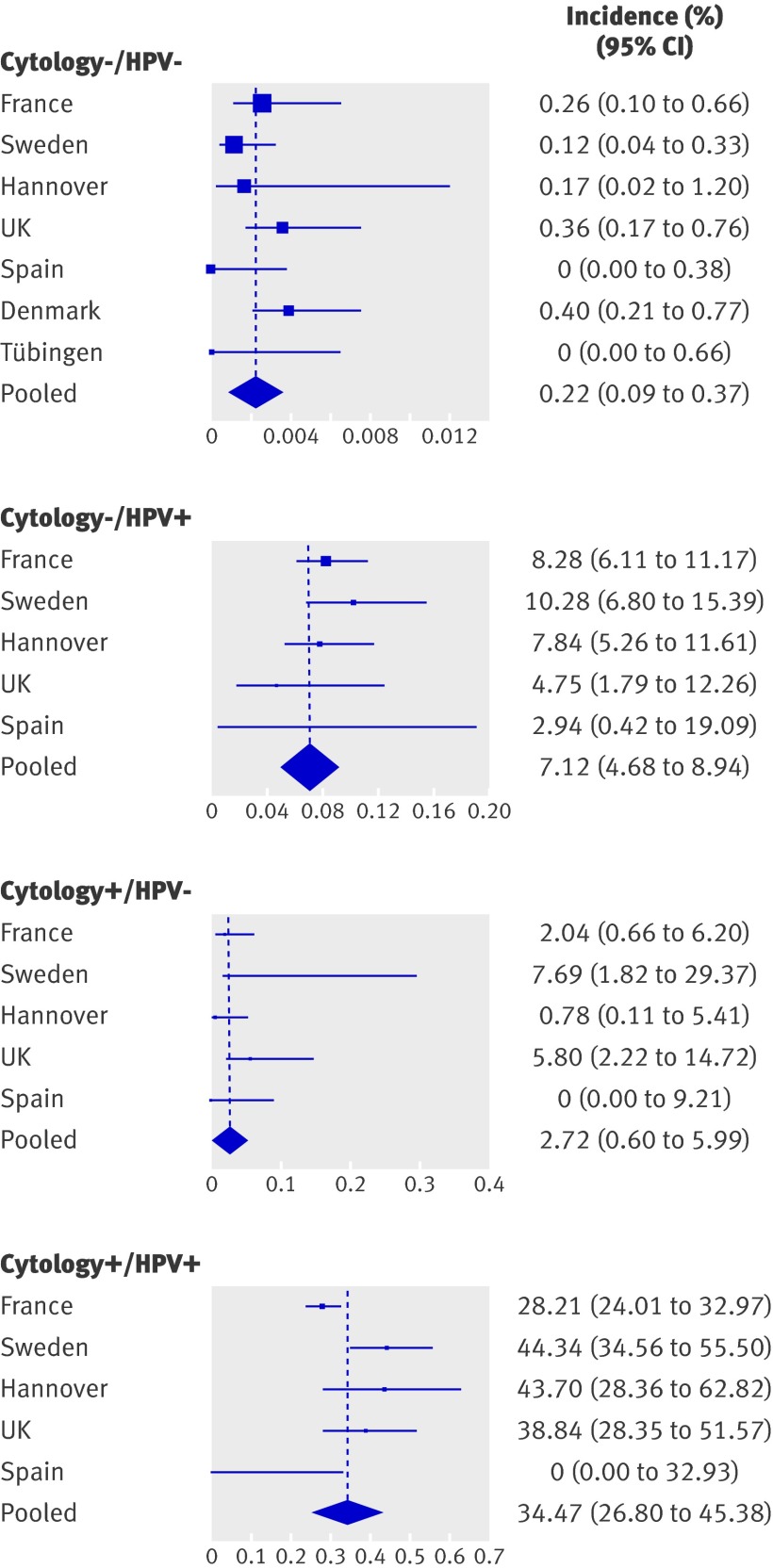
**Fig 4** Comparison of cumulative incidence rate (95% confidence interval) of CIN3+ at 60 months by country and by baseline test results

## Discussion

### Main findings and strengths

Using pooled data from seven HPV screening studies in six European countries we estimated a cumulative incidence rate for future histologically confirmed CIN3+ during six years of follow-up. The uniformly low rate among women with negative results on both cytology and HPV tests suggests that double negativity confers a long lasting protective effect that is remarkably robust, considering that the participating studies used several different types of HPV tests in several different settings and in several different age groups. The long lasting protective effect was similarly low in women negative for HPV and women with negative results on both tests.

That several studies in different settings in different countries and with different infrastructure and intensity of follow-up gave largely similar results is a strength of the study, as it implies that the data are generalisable to various settings. Similarly, that we studied the actual cytological tests used in the different countries implies that the data are generalisable—for example, the largest study in the joint cohort (France) used the most modern cytology technique (liquid based cytology) and several other cohorts used the same routine conventional cytology as is used in organised programmes with a documented preventive effect on cancer.

### Consistency with other studies

Our results agree with the results from a US cohort of 20 810 women that found that cytology−/HPV− women had a cumulative incidence rate of CIN3+ of 0.16% after 45 months and 0.79% after 122 months.^7^ Similarly, in a German cohort of 4034 women, 0.7% of cytology−/HPV− women developed CIN3+ during five years of follow-up^8^ and in a Dutch cohort of 2810 women there was only one case of CIN3+ among women with negative results on both tests during 4.6 years of follow-up.^28^

### Limitations and other considerations

As expected, the HPV test was less specific than cytology. The higher specificity in women aged over 35 suggests that restricting HPV testing to older women would reduce overdiagnosis. With increasing length of follow-up, however, the cumulative incidence rate for CIN3+ increased more among women positive for HPV than among women with positive cytology results. This implies that the problem of HPV based screening resulting in increased overdiagnosis, with women unnecessarily referred for clinical procedures, is attenuated in evaluations with longer follow-up—some of the HPV positivity that seems to be false positivity in cross sectional evaluations will turn out to be true, but earlier, detection of CIN3+.

Verification bias might overestimate the performance of screening tests when only women with a positive screening test result are referred for colposcopy. Only some of the included studies performed colposcopies in women with negative results on both tests. It is, however, rare to diagnose CIN3+ by colposcopy in such women^29^ ^30^ and the fact that there was limited variability between studies also suggests that verification bias has not materially affected our estimates.

Nevertheless, as assessment of the incidence of CIN3+ by baseline group during follow-up depends on the intensity of screening our estimates should be interpreted as relative rather than absolute.^31^ We included only women who had been screened at least once during follow-up, and the follow-up time was longer than the recommended screening intervals in all the included countries.^32^

In one study (Spain), no action was taken because of positive results of HPV tests at baseline whereas four studies mandated extra testing or colposcopy, or both (Sweden, Hannover, UK, and France). As we did not include Denmark and Tübingen in the follow-up of women with positive results on cytology or HPV tests, our cumulative incidence rate of CIN3+ is almost entirely based on active follow-up of HPV tests and should reflect the outcome of active HPV based screening strategies.

As prevalence of CIN3+ is associated with prevalence of HPV some heterogeneity between studies might be explained by differences in prevalence of HPV—for example, Spain has a low prevalence of HPV.^22^ Another possible source of variability is the fact that the German, French, Danish, British, and Spanish studies used hybrid capture II for HPV detection, while the Swedish study used polymerase chain reaction. The agreement between the two is substantial,^33^ ^34^ and there was no obvious difference in results depending on the HPV test used. The most obvious source of heterogeneity between countries was the variability in interpretation of cervical smear tests, as the proportions of positive cytology results ranged from 2% in Sweden to over 4% in Hannover and Spain, 5% in the UK, and 7% in France; these differences cannot be entirely explained by the observed differences in the prevalence of HPV.

In conclusion, these joint European data suggest that screening intervals could safely be lengthened to six years among women with a negative result on an HPV test. This could at least partly compensate for the increased referral rate resulting from HPV based screening strategies.

**What is already known on this topic**

Cervical screening with HPV testing is more sensitive for detection of cervical intraepithelial neoplasia grade 3 or cervical cancer (CIN3+)
Cost efficiencies of cervical screening strategies combining cytology with HPV testing depend on the duration of the protective effect of negative testing as this determines the optimal screening interval

**What this study adds**

A joint analysis of seven different studies in six European countries consistently found a low six year cumulative incidence rate of CIN3+ among women negative for HPV
Cervical screening strategies with HPV testing every six years is safe and effective

## Notes

We thank Caspar Looman for statistical advice and all participating patients and the gynaecologists and midwives who helped out with enrolment or follow-up of the cohort; Michael Menton and Annette Stubner for enrolment in Tübingen; C Coll, R M Vilamala, M Badia, R Font, F Martinez, A Avecilla, A Ramirez, H Mausbach, F J García, C I Rera, R Bosser, M P Cañadas, P Felipe, R Almirall, M Olivera, and J Klaustermeiyer for enrolment in Spain; B G Hansson, W Ryd, A Strand, G Wadell, E Rylander, and S Törnberg (steering group members, Sweden); and C Quereux, O Graesslin, J P Bory, C Pia, and M F Poncelet for enrolment in France.

Contributors: PB and CC (France); K-UP and TI (Germany); AS and JC (UK); SdeS and BL (Spain); SK and CM (Denmark); JD and PN (Sweden) were the principal investigators or coprincipal investigators and designed or coordinated the study in each country. TI coordinated the joint analysis project. The joint database was established and primarily analysed by MR under the supervision of MvB and JD. JD, PN, and MR primarily drafted the manuscript, to which all authors contributed. JD is guarantor.

Provenance and peer review: Not commissioned; externally peer reviewed.

## Notes

**Cite this as: ***BMJ* 2008;377:a1754
